# Orthodontics problems in pediatric and growing subjects

**DOI:** 10.1186/1824-7288-40-S1-A74

**Published:** 2014-08-11

**Authors:** Paola Cozza, Roberta Lione

**Affiliations:** 1 Department of Clinical Sciences and Translation Medicine, University "Tor Vergata", Rome, 00133, Italy

## Background

In literature, the most effective timing for intercepting skeletal malocclusion is still widely debated. The aim of this study was to analyze the main orthodontic and occlusal problems needing correction in growing patients.

## Materials and methods

Crossbite, open bite and Class III malocclusion were observed and compared each one with their own control groups with the same age and dentoskeletal characteristics. The statistical comparisons between the test and control groups were performed with independent sample t tests and chi-square tests (P <.05).

## Results

The presence of posterior crossbite was significantly greater in growing subjects with oral breathing thus confirming the influence of these factors on skeletal development with constriction of the whole palate. Unilateral posterior crossbite is often associated with mandibular lateral displacement that is clinically characterized by deviation of the chin, facial asymmetry, dental midline discrepancy, and high prevalence of internal derangement of the temporomandibular joint. Children with anterior openbite presented with a greater prevalence rate of sucking habits. Hyperdivergency is a risk factors for negative overbite in mixed dentition subjects. The test group had significantly smaller maxillary intermolar and intercanine widths and greater posterior transverse discrepancy. Class III malocclusion is a complex clinical entity that entail the contraction of the maxilla and a narrowing of the base of the nose in addition to an increased mandibular total length.

## Conclusions

Treatment of orthodontic problems that do not improve with age may be started earlier to avoid worsening of the condition in permanent dentition. The objective of any treatment in pediatric subjects before eruption of all permanent teeth are to correct the skeletal discrepancy between the jaws and improve function and facial esthetics by allowing them to develop normally, to create an ideal overbite and overjet relationship, to align the anterior permanent teeth and reduce the chance of trauma to these teeth, to improve the width of the dental arches and to reduce the risk for extraction of permanent teeth and for surgery in severe cases.

Morphologic and functional characteristics of unilateral posterior crossbite with mandibular lateral deviation should be clarified to correct and prevent this malocclusion. Children with mouth-breathing pattern and sucking habits showed a significant constriction of the maxillary arch and an increased palatal height when compared with control group. Early treatment of Class III malocclusion is able to produce significant and favorable long-term skeletal shape changes characterized by an anterior morphogenetic rotation of the mandible.

